# On the Treatment of Typhoid Fever by Cold Water Baths

**Published:** 1871-04

**Authors:** J. H. Tyndale

**Affiliations:** House Surgeon to the German Hospital, N. Y.


### On The Treatment of Typhoid Fever by Cold Water Baths.

By J. H. Tyndale, M. D., House Surgeon to the German Hospital, N. Y.

Late researches have developed a method of cure, by the application of which we are enabled to reduce the rate of mortality among typhoid fever patients by from three to five per cent. This method is the treatment of typhoid fever by cold water baths, practiced in the last century, but lately revived by Brand, of Stettin, and since submitted to a scientific and practical test by a great number of physicians on the continent of Europe. The verdict in favor of this method of treatment of typhoid fever on rational principles has been universal, and attested by numerous and responsible clinical reports, comprising many thousands of cases.

The cold water treatment cannot prevent the natural course of typhoid fever. The natural phases, with their peculiar anatomical changes, will appear in an undiminished degree. Cases of death from perforation of the bowel or hemorrhage have not been diminished any more than if no treatment at all had been prescribed. The cases of death from these causes, however, has always been counted as an incomparably small fragment of the total mortality among typhoid fever patients. The principal source of danger for the patient is the fever heat, either directly or indirectly, and we are enabled to reduce this unnatural elevation of the temperature of the body, and thereby the degree of danger to the patient, by cold baths. The dangerous effect of fever heat in typhoid fever, as manifested by the so-called nervous manifestations: delirium, sleeplessness in the first stage, listlessness in the second course of the disease depends entirely upon the continued unnatural temperature of the body. Death from these causes has never revealed any anatomical lesions. Such symptoms will not manifest themselves if by timely, energetic, and oft-repeated withdrawal of warmth, a cooling off of the body is effected. The patient remains sensible, and involuntary voiding of fæces and urine is of rare occurrence.

Not merely the psychical manifestations, but also the other functions of the nervous system, as well as muscular activity, are more or less impaired by fever heat. The paralyzing influence is shown by the feebleness of respiration, giving rise to the fatal collapse and inflammatory conditions of the lungs. The fever heat checks or limits excretion, interferes in this way with the functions of digestion, produces loss of appetite, and stops the supply of natural material necessary to supply the rapid waste of tissue.

All these sequels of the feverish overheating of the body are observed to manifest themselves in a lesser degree by the use of cold water baths. Thus, all competent observers are agreed upon the fact that the patient never loses his appetite; on the contrary, takes food during the whole course of the fever, so that extreme emaciation will not ensue, and the patient regains his strength in a shorter space of time from the period of convalescence. Bed sores, so frequent and unavoidable in typhoid fever, have been but rarely observed during the cold water treatment. In short, all secondary complications of typhoid fever have been totally excluded by this method of treatment, and the whole course of the disease has been completed in four weeks.

The general rules to be observed in administering cold water baths are the following:

1. The necessary reduction of temperature is best and most rapidly effected by immersing the whole body.

2. The water should be as cold as can be had.

3. The patient should be bathed as often as the temperature of his body, measured in the rectum, rise to 40° C. [about 104° Fahr.] Since the intensity of the manifestations of disease vary much, it may occur that in one case one or two baths in the twenty-four hours will suffice, whereas, in another, as many as twelve or sixteen will be required in the same space of time.

4. The length of time for each bath must be governed on the one hand by the degree of fever heat, on the other hand by the temperature of the water used. On the whole it will be found that in a bath varying from 5° to 10° C., an immersion of from seven to ten minutes will suffice. Should the temperature of the water be above 10° C., the bath is to be continued for fifteen minutes, and if above 15° C., still longer.

No attention needs to be paid to the seeming discomfort of the patient, manifested by complaints, nor to the chill often occurring during the bath, and continuing for sometime afterward, as they are of no consequence.

5. After the bath, the patient should be carefully wiped dry [not rubbed], especially his feet and toes. If the water has been of very low temperature, the feet may be enveloped in warm cloths, as many patients may complain of pain in the feet after a very cold bath.

Opinions differ as to whether it is best to immerse the patient in a cold bath, [say 10° C.] at once, or to have the temperature more nearly the same as that of the body, and effect a gradual reduction by a slow addition of cold water. Niemeyer, who may be considered the best authority upon the subject, is in favor of a gradual reduction. With due deference to this opinion, however, I must say that repeated trials have satisfied me that by a sudden immersion in cold water two advantages are gained—1st, the reduction of temperature will be greater, more nearly approximating the normal temperature of the body; 2d, less time will be required, and consequently the patient will be less annoyed. In the cases under our observation we have found from one-half to two hours after sudden immersion the temperature reduced to 38.5° C. [normal], when before the bath it had been from 40° to 40.5° C.

When the temperature of the body has not been above 39.5° C., we have been in the habit of enveloping the patient in wet cold sheets for fifteen minutes. In other cases in which it was desirable to move the patient as little as possible, we have resorted to a sponge-bath of cold water and vinegar. Both methods produce a limited decrease of temperature, not exceeding one degree.

The *thermometer* is indispensable as an aid to the cold water treatment, as without it this method would lack the necessary safety in its application. The rectum is undoubtedly the best point of observation of the thermometer. In five minutes after the introduction of the bulb, the mercury will have reached its maximum height, and no disturbing influence can injure the correctness of observation, as is often the case in the introduction of the bulb into the axilla.

The severer the case, the oftener should thermometrical observations be made. In mild cases, in which even the evening temperature [always higher than the morning temperature] does not exceed 40° C., two or three observations may suffice; whereas, in severer ones, this should be done every two hours day and night, in order not to miss the right time for the repetition of the bath. —*St. Louis Medical and Surgical Journal.*

